# Performance comparison of next-generation sequencing platforms for determining HIV-1 coreceptor use

**DOI:** 10.1038/srep42215

**Published:** 2017-02-10

**Authors:** Stéphanie Raymond, Florence Nicot, Nicolas Jeanne, Olivier Delfour, Romain Carcenac, Caroline Lefebvre, Michelle Cazabat, Karine Sauné, Pierre Delobel, Jacques Izopet

**Affiliations:** 1 INSERM, U1043, Toulouse, F-31300, France; 2 Université Toulouse III Paul-Sabatier, Faculté de Médecine Toulouse-Purpan, Toulouse, F-31300, France; 3 CHU de Toulouse, Hôpital Purpan, Laboratoire de Virologie, Toulouse, F-31300, France; 4 CHU de Toulouse, Hôpital Purpan, Service des Maladies Infectieuses et Tropicales, Toulouse, F-31300, France

## Abstract

The coreceptor used by HIV-1 must be determined before a CCR5 antagonist, part of the arsenal of antiretroviral drugs, is prescribed because viruses that enter cells using the CXCR4 coreceptor are responsible for treatment failure. HIV-1 tropism is also correlated with disease progression and so must be determined for virological studies. Tropism can be determined by next-generation sequencing (NGS), but not all of these new technologies have been fully validated for use in clinical practice. The Illumina NGS technology is used in many laboratories but its ability to predict HIV-1 tropism has not been evaluated while the 454 GS-Junior (Roche) is used for routine diagnosis. The genotypic prediction of HIV-1 tropism is based on sequencing the V3 region and interpreting the results with an appropriate algorithm. We compared the performances of the MiSeq (Illumina) and 454 GS-Junior (Roche) systems with a reference phenotypic assay. We used clinical samples for the NGS tropism predictions and assessed their ability to quantify CXCR4-using variants. The data show that the Illumina platform can be used to detect minor CXCR4-using variants in clinical practice but technical optimization are needed to improve quantification.

Human immunodeficiency virus (HIV) enters its host cells following the interaction between the virus envelope glycoprotein (Gp120), the cell surface CD4 receptor, and a chemokine receptor, which may be CCR5 and/or CXCR4, acting as a coreceptor^1^. The HIV tropism is defined by the coreceptor(s) use and is correlated with disease progression^2^. Thus, it is essential to determine the HIV coreceptor used before including a CCR5 antagonist in a patient’s antiretroviral regimen^3^. Previous clinical studies have found that minor variants in the virus quasi-species may be responsible for the virological failure of a CCR5-antagonist-based treatment^4^,^5^. Deep sequencing techniques can detect minor variants, especially ones that use CXCR4^6^,^7^,^8^. The 454 GS-Junior sequencing platform (Roche) can reliably predict HIV-1 tropism^9^,^10^,^11^. The Ion Torrent Personal Genome Machine has also been validated for tropism determination^12^. Another study evaluated the Illumina platform for determining HIV-1 drug resistance and HIV-1 tropism^13^,^14^,^15^. Nevertheless, analytical validation for determining HIV-1 tropism in clinical practice is still limited. We, therefore, evaluated the performances of the Illumina MiSeq platform for predicting HIV tropism by comparing its performance with that of the 454 GS-Junior system and a reference recombinant virus phenotypic entry assay.

## Results

### Sensitivity of deep sequencing for detecting minor CXCR4-using variants

**Figure 1:**
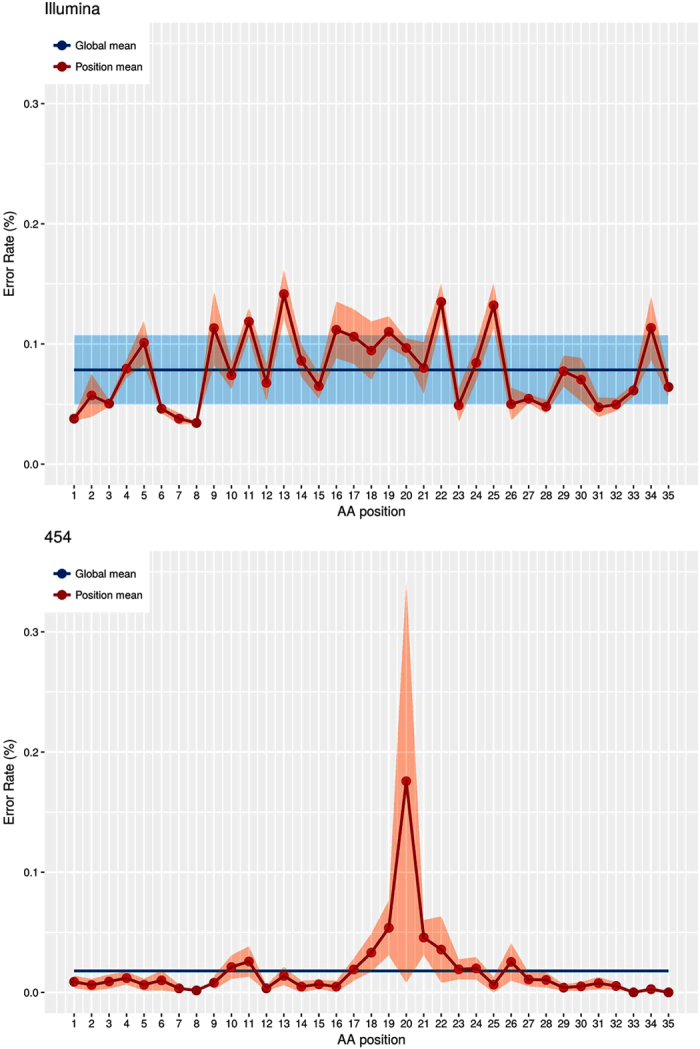
Error rate of amplification and deep sequencing for each NGS platform at each position of the V3 sequence. The error rates for the MiSeq Illumina (Fig. 1A) and 454 GS-Junior (Fig. 1B) are shown on two separate graphs. The global mean (blue line) is the mean frequency of artifactual V3 variants of 20 virus clones. The position mean (red line) is the error rate estimated at each position of V3 by comparing the UDS reads to the Sanger sequences of 20 clones. The shaded regions represent the 99% confidence interval of global (blue) and position (red) mean error rates.

We determined the sensitivity threshold of NGS by calculating the error rate, either globally or for each position of V3. The mean frequency of V3 variant artifacts determined after analysis of 20 virus clones with the Illumina was 0.078% [exact Poisson 99% confidence interval (CI), 0.059–0.097] of the reads (Fig. 1). This fixed cut-off provided sensitivity thresholds of 0.35% for 2000 reads of MiSeq and of 0.26% for 5000 reads. We then used the same material to compare the systems. The mean frequency of V3 variant artifacts found with the 454 GS-Junior was 0.018% [exact Poisson 99% CI, −0.033–0.039] of the reads. This provided a sensitivity threshold of 0.25% for 2000 reads with this system. We next determined position-specific error rates along the V3 sequence, defined as the upper 99% confidence interval (Poisson statistics) of the mean frequency of artifactual codons at each V3 position among the 20 virus clones (Fig. 1). We then attributed a weighted error rate to each position and used these rates to construct a sensitivity threshold matrix for each position of V3 to retain a minor virus variant harboring point mutations as authentic for a given number of reads with P < 0.001. The position-specific mean error rate of the MiSeq system was 0.034% at position 8 of V3 and 0.142% at position 13 V3, with great variation over the full length of the V3 region. The position-specific mean error rate of the 454 GS-Junior system was 0% at positions 33 and 35 and 0.176% at position 20 of V3. The largest variations occurred between amino acids 19 and 21, which are located in a homopolymeric region.

### Sensitivity and linearity for quantifying CXCR4-using variants

**Figure 2:**
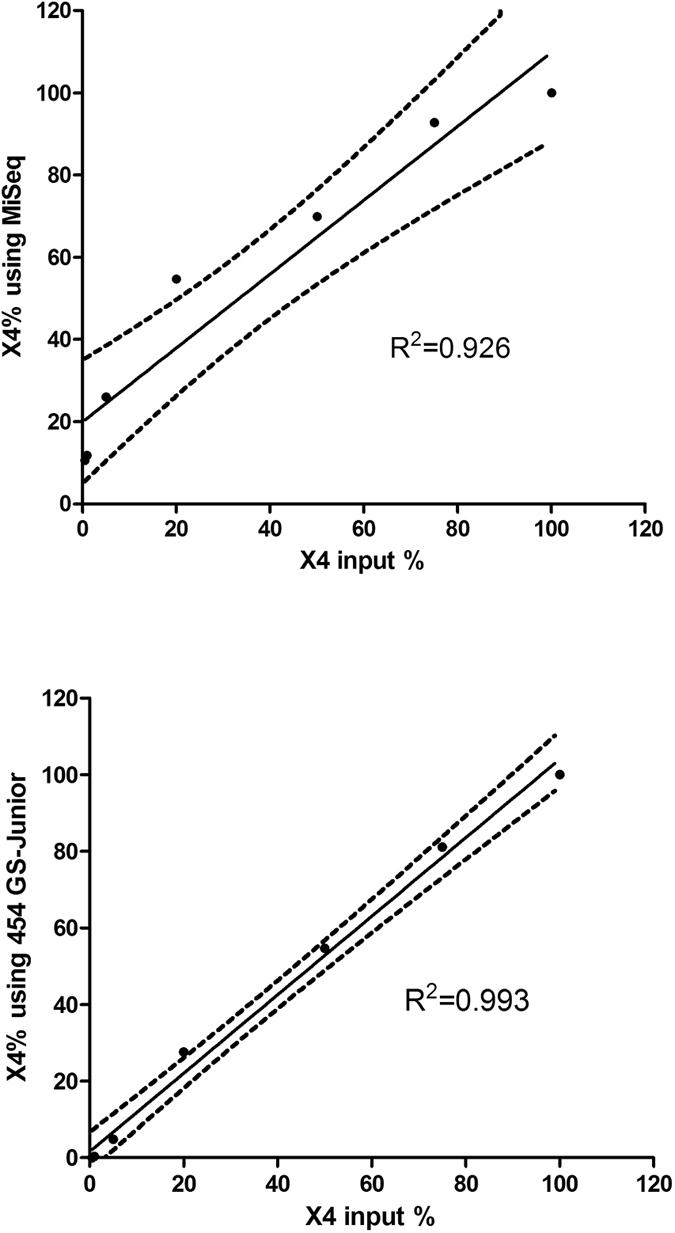
Quantifying X4 variants in HIV-1 quasispecies by deep sequencing. Each CHS11(R5):CHS02 (X4) virus mixture was assessed in triplicate or duplicate beyond 5%. Figure 2A. Linear regression of the percentage of X4 variants quantified by MiSeq. Figure 2B. Linear regression of the percentage of X4 variants quantified by 454 GS-Junior. Solid black line is the regression line. Dotted black lines show the 95% confidence interval of the mean.

We evaluated the sensitivity of the NGS for quantifying CXCR4-using variants with artificial mixtures of pure X4 (CHS02) and R5 (CHS11) virus clones. The MiSeq NGS detected 0.5% of X4 viruses in 2/3 mixtures and 1% in 3/3 mixtures. The 454 GS-Junior NGS detected 1% of X4 viruses in 2/3 mixtures and 5% of X4 viruses in 3/3 mixtures (Table 1). Linear regression analysis of the proportion of CXCR4-using variants (0.5 to 100%) plotted against the expected frequencies yielded coefficients of determination of R^2^ = 0.926 for the MiSeq data (Fig. 2A) and R^2^ = 0.993 for the 454 GS-Junior data (Fig. 2B). Spearman analysis showed significant correlations between the expected values and the values obtained with both MiSeq (ρ = 1, p < 0.001) and the 454 GS-Junior (ρ = 1, p < 0.001). Linear regression showed that the MiSeq system overestimated the X4 variants (MiSeq = 19.95 + 0.90*X4 input) while the proportion of X4 variants quantified with 454 GS-Junior was very similar to the theoretical value (454 GS-Junior = 1.58 + 1.02*X4 input).

### Comparison of deep sequencing and a phenotypic method for predicting tropism

We compared the genotypic predictions obtained using the MiSeq and 454 GS-Junior systems and the Pyrovir tropism prediction (http://diag.ablsa.com/pyrovir/submit.php) and the phenotype using TTT in 52 plasma samples from antiretroviral naïve patients (Table 2). The phenotypic assay found 33 R5 samples and 19 R5X4/X4 samples. The MiSeq and Pyrovir methods correctly predicted 30 R5 samples and 10 X4 samples with an overall concordance with the phenotype of 77% (Table 3). The 454 GS-Junior and Pyrovir systems correctly predicted 28 R5 samples and 9 X4 samples with an overall concordance with the phenotype of 71%.

The concordance between the two NGS methods for predicting HIV-1 tropism was 90%. The two NGS methods disagreed on 5 samples (samples 29, 30, 41, 45 and 48 in Table 2): MiSeq predicted two to be X4-using (X4 variant frequencies: 0.3 and 0.7%) that were R5 according to the 454 GS-Junior. The 454 GS-Junior predicted 3 samples as X4 (X4 variant frequencies: 0.2, 0.7 and 8.7%) while the MiSeq system identified them as R5. The phenotype of the discordant samples showed that MiSeq correctly predicted the tropism of four of the five samples (samples 29, 30, 41 and 45).

We also evaluated the capacity of the MiSeq and 454 GS-Junior systems to accurately quantify CXCR4-using variants by comparing the percentages of X4 variants in 11 samples with concordant detection of X4 variants (Table 2). The MiSeq system gave the percentage of CXCR4-using variants as 1.3–100% (mean = 45.2%), while the 454 GS-Junior system gave values of 2.6–100% (mean = 60.1%). Spearman analysis showed a significant correlation between the two methods (Spearman, ρ = 0.667, p = 0.025).

## Discussion

We assessed the analytical performance of the MiSeq Illumina deep sequencing system for determining HIV-1 coreceptor use and compared it to that of the 454 GS-Junior Roche method. The capacities of the two deep sequencing methods for determining HIV-1 tropism in clinical samples were also compared to the reference phenotypic method.

We first determined the error rate of the NGS resulting from PCR bias and sequencing errors. The error rate of the MiSeq Illumina (0.078%) was greater than that of the 454 GS-Junior Roche (0.018%), but this was partly offset by more reads (mean: 5542) with the MiSeq than with the 454 GS-Junior (mean: 3480). We used the error rate to discriminate between the true and artifactual mutations^9^. The sensitivities of the two systems for detecting minor CXCR4-using variants were similar: 0.26% for 5000 reads for the MiSeq Illumina and 0.25% for 2000 reads for the 454 GS-Junior Roche. Our results for the Illumina deep sequencing are thus similar to those of another study that found error rates of 0.47% for the HIV reverse transcriptase plasmids and of 0.26% for the HCV NS3 plasmids at nucleotide level^16^. The position-specific error rate obtained using MiSeq was homogeneous because the nucleic acid combination does not influence the errors of PCR or sequencing. In contrast, the pyrosequencing was influenced by the presence of homopolymeric regions as previously described^17^.

Previous studies have shown that minor CXCR4-using variants are responsible for the failure of CCR5-antagonist based treatments^5^,^6^. Thus, assays that are very sensitive for detecting minor variants are required to ensure the safe use of CCR5 antagonists. We used mixtures of R5 and X4 virus clones to determine the sensitivities for detecting minor X4 variants. The MiSeq system was more sensitive (0.5–1%) than the 454 GS-Junior system (1–5%), perhaps because more reads were obtained with the MiSeq than with the 454 GS-Junior. This sensitivity is similar to that published for the 454 GS-Junior^10^. The quantification of X4 variants in the mixtures showed that the MiSeq overestimated the frequency of X4 viruses by around 20% for low values. As we mentioned in Methods, the PCR protocols used on the two platforms were slightly different, which could explain how PCR bias may have favoured the amplification of X4 clones of CHS02 strain. Thus, technical optimization is required to improve the accuracy of CXCR4-using variant quantification, as was done for the 454 GS-Junior assay^9^. However, the CXCR4-using variants in the clinical samples quantified using the MiSeq system correlated well with the 454 GS-Junior data.

The two NGS assays agreed in their analyses of 90% of the 52 clinical samples, similar to the concordance between two phenotypic assays for predicting HIV-1 tropism^7^,^18^. The concordance of both NGS assays with the phenotypic assay was higher than 70%. However, genotypic prediction using deep sequencing was less sensitive for detecting X4 viruses compared to a phenotypic assay. The genotypic prediction is often poor for the non-B subtypes of HIV-1^19^. However, the 52 samples in our panel included only four samples with non-B subtypes (2 CRF01-AE, 1 CRF02-AG and 1 subtype C) and a correct prediction was observed for two samples (1 CRF01-AE and 1 subtype C). We also tested the capacity of two other genotypic algorithms, the CM classifier (http://spg.med.tsinghua.edu.cn/CM.html) and geno2pheno 5.75% (http://coreceptor.geno2pheno.org/index.php) for predicting tropism^20^,^21^. The sensitivity of the CM classifier for detecting the CXCR4-using variants was only 42% and 53% using the data set obtained with the MiSeq and 454 GS-Junior, respectively (data not shown). The sensitivity of geno2pheno 5.75% for detecting the CXCR4-using variants was 63% using both data sets and the difference with the sensitivity of Pyrovir was not significant (data not shown). Thus, the performance of Pyrovir for predicting tropism using deep sequencing was similar to the performance of other validated genotypic algorithms on this data set. The sensitivity of the genotypic algorithms needs to be improved for the prediction of CXCR4-using variants. Only the V3 region of the envelope is used to predict HIV-1 tropism while the complete envelope sequence is used in the phenotypic assay. Previous studies have shown that other envelope regions are involved in the coreceptor used for virus entry^22^,^23^. New generations of sequencing systems that allow sequencing of the complete HIV-1 envelope and new algorithms based on analysis of other regions may improve the genotypic prediction of HIV-1 coreceptor use.

We have demonstrated that the MiSeq Illumina technology is as accurate as the 454 GS-Junior Roche system technology for determining HIV-1 tropism compared to a reference phenotypic assay. However, the quantification of CXCR4-using variants needs to be improved. Next-generation sequencing plus accurate genotypic algorithms for predicting HIV-1 tropism will eventually provide sensitive methods for managing the administration of CCR5 antagonists and for pathophysiological studies.

## Materials and Methods

### Samples and nucleic acid extraction

Plasma samples were obtained from 52 HIV-1-infected patients followed at the Department of Infectious Diseases of Toulouse University Hospital, France. Their HIV-1 tropism was determined during follow-up in accordance with French medical guidelines. The experimental protocol was approved by the Toulouse University Hospital. The patients gave their informed consent for virological studies. Plasmid clones of *env* obtained from HIV-1 subtype B primary isolates were used to determine the error rates of the deep sequencing systems. Artificial mixtures of X4 and R5 virus clones were used to assess the sensitivity and linearity of assays of minor CXCR4-using variant by these systems. CHS02 (GenBank accession no. DQ136867.1, X4 phenotype) and CHS11 (GenBank accession no. DQ136859.1, R5 phenotype) were mixed in defined proportions of X4:R5 viruses (0:100; 0.5:99.5; 1;99; 5:95; 20:80; 50:50; 75:25 and 100:0, each with 2–3 replicates). Virus RNA was extracted using the QIAamp Viral RNA Mini Kit.

### Phenotypic characterization of HIV-1 coreceptor usage

We determined the HIV-1 tropism with the Toulouse Tropism Test (TTT) phenotypic assay^24^. Briefly, a fragment encoding the gp120 and the ectodomain of gp41 was amplified by reverse transcription-PCR (RT-PCR) using HIV-1 RNA isolated from the patient’s plasma. The PCR products were then subjected to nested PCR. Two amplifications were performed in parallel on aliquots of each sample; the amplified products were then pooled to prevent sampling bias of the virus population. The phenotype of HIV-1 coreceptor use was determined using a recombinant virus entry assay with the pNL43-Δenv-Luc2 vector. We transfected 293 T cells with both the NheI-linearized pNL43-Δenv-Luc2 vector DNA and the product of the nested PCR obtained from the challenged HIV-1-containing sample. The chimeric recombinant virus particles released into the supernatant were used to infect U87 indicator cells bearing CD4 and either CCR5 or CXCR4. Virus entry was assessed by measuring the luciferase activity in lysed cells. Minor X4 variants were detected when they accounted for 0.5% or more of the total population as assessed in experiments performed on mixtures of various X4/R5 DNA ratios.

### 454 GS-Junior/Roche deep sequencing of the HIV-1 V3 env region

A 415-nucleotide long fragment encompassing the V3 *env* region was generated by nested PCR as previously described^9^. The amplified PCR products were purified using Agencourt Ampure PCR purification beads (Beckman Coulter, Brea, CA) and quantified using a Quant-iT Picogreen dsDNA Assay Kit (Invitrogen) on a LightCycler 480 (Roche Diagnostics, Meylan, France). PCR amplicons were combined and clonally amplified on DNA capture beads in water-in-oil emulsion micro-reactors. A total of 500,000 DNA-enriched beads were deposited in the wells of a full GS Junior Titanium PicoTiterPlate device and pyrosequenced in both the forward and reverse directions. The sequences of the V3 env regions were first processed using GS Amplicon Variant Analyzer (AVA) software (Roche). Only sequences that had been read in both senses were used for further analyses.

### Illumina deep sequencing of the HIV-1 V3 env region

The samples were prepared for sequencing on Illumina MiSeq at the genomic platform of Toulouse (http://get.genotoul.fr/). The first PCR amplified, in duplicate, a 3075-nucleotide long fragment. The nested PCR amplified a 406-nucleotide long fragment encompassing the V3 env region. The amplified PCR products were purified using Agencourt Ampure PCR Purification beads (Beckman Coulter, Brea, CA) and quantified using a Quant-iT Picogreen dsDNA Assay Kit (Invitrogen) on a LightCycler 480 (Roche Diagnostics, Meylan, France). PCR amplicons were combined and diluted to obtain a concentration of 30 ng/μL and the Nextera XT DNA Library Preparation kit was used for index amplification. Reads containing undefined nucleotides (‘N’s) were filtered out. This can occur when the signals of nucleotides incorporated in a cluster are much lower than those of surrounding clusters. Only sequences with an average phred equivalent quality score of >Q30 were retained.

### Genotypic prediction of HIV-1 coreceptor use

The sequence reads were aligned with the BaL consensus sequence (GenBank accession no. AY426110.1) and processed using an in-house automated data cleaning strategy^9^. Non-functional V3 sequences were discarded using biological filters and statistical filters were used to discard sequences with artifactual point mutations. As previously reported^9^, V3 sequences were considered to be non-functional if (i) they did no start and end with the cysteine required for the disulfide bond; (ii) they were not 32–38 amino-acids long; (iii) they contained a stop codon; (iv) they were not sufficiently like the V3 consensus at three typical motifs: a “CTRP”-like signature at the N-terminus, a “GPGR”-like signature at the hairpin crown, or a “QAHC”-like signature at the C-terminus with an identity of 0.5 and 0.75 for substitution and insertion/deletion of an amino-acid at any of the three signature motifs. This biological and statistical data cleaning strategy has been integrated in a program (PyroVir, IDDN FR.001.160011.000.S.P.2012.000.31230, Inserm-Transfert) that provides a fast, automated position-specific process for inferring HIV-1 tropism from V3 env deep sequencing data with improved detection of minor variants. The genotypic rule used to predict CXCR4-usage can be changed according to the virus subtype, particularly for subtypes D and CRF01-AE for which we have developed specific algorithms^25^,^26^. We used the combined 11/25 and net charge rules to infer the tropism of each virus clone from the V3 amino acid sequence. It requires one of the following criteria for predicting the CXCR4 coreceptor usage of HIV-1 subtype B^27^,^28^: (i) an R or K at position 11 of V3 and/or a K at position 25; (ii) an R at position 25 of V3 and a net charge of at least +5; and (iii) a net charge of at least +6. The V3 net charge was calculated by subtracting the number of negatively charged amino acids (D and E) from the number of positively charged ones (K and R). The CM classifier (generic prediction) was used to predict tropism with cleaned data of V3 amino acid sequences^20^.

### Determining the position-specific error rates of deep sequencing along the V3 sequence

As the errors generated during deep sequencing are sequence-dependent, we determined specific error rates at each position in V3. We measured the mean codon error rate, based on the worst error rate of the 3 nucleotides, among the 20 clones at each V3 position. The position-specific error rates were then defined as the upper limit of the 99% confidence interval of the mean frequency of artifactual codons among the 20 clones at each position of V3. We then determined weighted error rates to construct a sensitivity threshold matrix at each position of V3 to identify authentic virus variants harboring a point mutation for a given number of reads with P < 0.001 (Poisson statistics).

### Data analysis

Poisson statistics were calculated using R version 3.0.0. The linearity of the X4 variant quantifications was estimated by linear regression of data for mixtures of X4:R5 viruses (XLSTAT, Addinsoft). A Bland-Altman analysis (scatter plot of the differences between the paired measurements plotted against their means) of samples containing X4 variants obtained with MiSeq and 454 GS-Junior systems was used to assess graphically the magnitude of disagreement between the methods and estimate the overall bias. A Spearman’s correlation coefficient was used to assess the strength of linear association between the methods for quantifying X4 variants.

## Additional Information

**How to cite this article**: Raymond, S. *et al*. Performance comparison of next-generation sequencing platforms for determining HIV-1 coreceptor use. *Sci. Rep.* **7**, 42215; doi: 10.1038/srep42215 (2017).

**Publisher's note:** Springer Nature remains neutral with regard to jurisdictional claims in published maps and institutional affiliations.

## Figures and Tables

**Table 1:** Detecting and quantifying X4 variants in HIV-1 quasispecies by deep sequencing.

X4 input %	MiSeq			Mean	CV %	454 GS-Junior				CV %
	T1	T2	T3			T1	T2	T3	Mean	
0.5	0.0	10.7	21.0	10.6	99	0.0	0.0	0.0	0.0	-
1.0	17.6	15.0	2.9	11.8	66	0.6	0.5	0.0	0.4	88
5.0	18.3	35.2	24.5	26	33	5.0	4.2	5.3	4.8	12
20.0	51.9	57.4	—	54.7	7	30.0	25.2	—	27.6	12
50.0	61.1	78.7	—	69.9	18	56.2	53.1	—	54.7	4
75.0	96.0	89.6	—	92.8	5	80.6	81.6	—	81.1	1
100.0	100.0	100.0	—	100.0	0	100.0	100.0	—	100.0	0

Each CHS11(R5):CHS02 (X4) virus mixture was assessed in triplicate (T1, T2, T3) or duplicate beyond 5%.

**Table 2:** NGS and phenotypic assay results for 52 clinical samples.

Sample	HIV-1 subtype	Viral load (log cop/mL)	MiSeq		454 GS-Junior		Phenotype TTT
			Reads number	%X4	Reads number	%X4	
1	B	3.93	14779	<0.2	4964	<0.2	R5
2	B	5.32	7582	<0.2	4759	<0.2	R5
3	B	5.33	9402	<0.2	4765	<0.2	R5
4	B	3.89	5856	<0.2	5279	<0.2	R5
5	B	5.11	12150	<0.2	2415	<0.2	R5
6	B	6.70	4028	<0.3	2645	<0.2	R5
7	B	4.55	552	<0.6	3345	<0.2	R5
8	B	5.67	4766	<0.2	2948	<0.3	R5
9	B	4.89	6681	<0.2	5210	<0.3	R5
10	B	5.87	7131	<0.2	2675	<0.3	R5
11	B	6.11	12195	<0.2	977	<0.3	R5
12	B	5.82	3275	<0.3	1468	<0.3	R5
13	B	5.65	2534	<0.3	1606	<0.3	R5
14	B	5.03	2484	<0.3	2386	<0.3	R5
15	B	7.00	1997	<0.3	1331	<0.3	R5
16	B	7.00	3360	<0.3	1389	<0.3	R5
17	B	6.48	2378	<0.3	2793	<0.3	R5
18	B	6.72	2461	<0.3	2526	<0.3	R5
19	B	5.77	3221	<0.3	2541	<0.3	R5
20	B	5.83	1123	<0.4	1828	<0.3	R5
21	B	3.19	8817	<0.2	6513	<0.4	R5
22	C	5.13	3523	<0.3	1943	<0.4	R5
23	B	>7	10484	<0.2	2370	<0.5	R5
24	B	2.70	8379	<0.2	3478	<0.5	R5
25	B	4.89	1888	<0.3	1886	<0.5	R5
26	B	6.00	2456	<0.3	1719	<0.5	R5
27	B	6.95	2638	<0.3	629	<0.8	R5
28	B	5.47	1563	<0.4	180	<0.8	R5
29	B	5.81	10769	<0.2	4522	0.2	R5
30	B	4.66	5455	<0.2	4216	0.7	R5
31	B	5.81	5993	4.2	2246	2.6	R5
32	B	6.55	12181	3.3	2499	5.6	R5
33	B	7.00	4174	29.8	2383	9.5	R5
34	B	5.00	6983	7.3	2551	6	R5X4
35	B	5.90	2622	100	4291	100	R5X4
36	B	3.83	2366	100	2308	100	R5X4
37	CRF01	6.00	3640	<0.3	2881	<0.1	R5X4
38	B	6.34	5080	<0.2	2335	<0.3	R5X4
39	B	3.82	7560	<0.2	2851	<0.3	R5X4
40	B	4.34	3332	<0.3	5562	<0.4	R5X4
41	B	5.78	6053	0.3	3372	<0.4	R5X4
42	B	6.41	5333	<0.2	25085	<0.5	R5X4
43	B	3.83	4882	<0.2	3317	<0.5	R5X4
44	CRF02	5.58	2054	<0.3	4647	<0.5	R5X4
45	B	4.09	1948	0.7	5395	<0.5	R5X4
46	B	4.55	19404	<0.2	1950	<0.6	R5X4
47	B	4.46	948	40.3	3137	38.9	R5X4
48	B	4.75	4264	<0.3	2694	8.7	R5X4
49	B	2.78	3313	100	4120	99.4	R5X4
50	B	6.64	2657	1.3	3283	99.6	R5X4
51	CRF01	2.69	546	11.5	5330	99.7	R5X4
52	B	5.08	8977	100	2799	100	X4

**Table 3:** HIV-1 tropism predictions by the two NGS methods and a phenotypic method.

		Phenotype TTT		Concordance NGS/TTT
		R5	X4	
MiSeq/PyroVir	R5	30	9	77%
	X4	3	10	
454 GS-Junior/Pyrovir	R5	28	10	71%
	X4	5	9	
